# Extraction of dye sorghum biocolorant for the dyeing of *wagashi*, a West African soft cheese

**DOI:** 10.1016/j.heliyon.2024.e39065

**Published:** 2024-10-09

**Authors:** Oscar Bienvenu Oloudé Odouaro, Adéchola Pierre Polycarpe Kayodé, Médérice Sènanmi Behanzin, Martinus J. Rob Nout, Anita R. Linnemann

**Affiliations:** a Laboratory of Valorisation and Quality Management of Food Bio-Ingredients, Faculty of Agricultural Sciences, University of Abomey-Calavi, 01 BP 526, Cotonou, Benin; b Ronfostec, Papenpad 14, 6705 AX, Wageningen, the Netherlands; c Food Quality and Design, Wageningen University, P.O. Box 17, 6700 AA, Wageningen, the Netherlands

**Keywords:** *Sorghum bicolor*, Sorghum sheaths, Solvents, Biocolorant, *Wagashi*

## Abstract

We developed a sustainable method to extract the red biocolorant from dye sorghum leaf sheaths for dyeing *wagashi*, a West African soft cheese. The pigments were extracted using three different solvents: commercial ethanol (method A), *sodabi* (a local liquor distilled from palm wine; method B) and aqueous alkaline solvent (method C). In methods A and B, a pot still was used to distil and collect the solvent for reuse. The obtained extracts were used to dye *wagashi* at various concentrations. The yield and colour characteristics of the extracts were evaluated and the physicochemical characteristics of the dyed *wagashi* were recorded and compared to commercial *wagashi* sold in the local market. The solvent recovery in methods A and B was 96 % and 47 %, respectively. Extraction of anthocyanin pigments was highest with method A, namely 21 mg mL^−1^ for total phenolics against 13.4 and 1.64 mg mL^−1^ for methods B and C, respectively. The colour parameters suggested a similar colour for all the extracts, but the redness index of the extract from method B was significantly higher than that of the other methods. *Wagashi* dyed with extracts from methods B and C had similar colour characteristics and were comparable to the best-coloured commercial *wagashi* from the Parakou market based on the chroma and the hue angle values of the chromaticity diagram. Despite the lower colouring power of extracts made by method B, we consider this method the best when taking into account the cost and availability of the solvent. In conclusion, the application of *sodabi* extract at a dose of 200 mL L^−1^ is recommended to dye *wagashi*.

## 1 Introduction

**Fig. 1:**
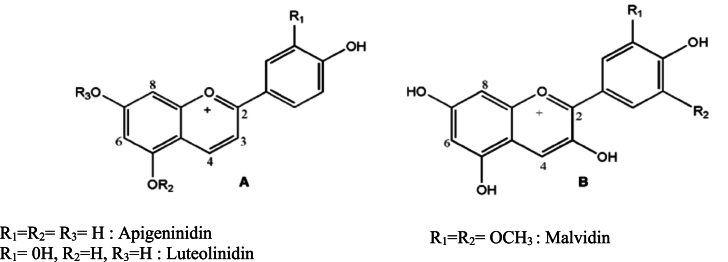
Structure of (A) the 3-deoxyanthocyanidins and (B) malvidin, a common anthocyanin.

Source: Awika et al. (2004).

Colour is a sensorial attribute that largely explains product perception by consumers [1]. Nowadays, consumers are increasingly interested in natural colourants because of concerns about synthetic alternatives [2,3]. The use of colourants from dye plants developed in Medieval times and the growing of dye plants reached its peak in the 18th century [4]. This development favoured the use of natural colourants as additives in food, pharmaceutical and cosmetic sectors. In the West Africa region, the leaf sheaths of a specific sorghum (*Sorghum bicolor*) variety, known as dye sorghum, are used to colour foods (e.g., local soft cheese known as *wagashi*, and porridge) and in traditional medicine [5,6]. The red pigment extracted from the leaf sheaths of dye sorghum is rich in 3-deoxyanthocyanidins, a rare class of natural pigments with health-promoting properties [5,7]. The 3-deoxyanthocyanidins exhibited high stability under processing conditions compared to other anthocyanins because they lack an oxygen atom at carbon 3 [7] (Fig. 1A and B). Yang, Browning and Awika [8] demonstrated that sorghum 3-deoxyanthocyanidins possess strong Phase II enzyme inducer activity and cancer cell growth inhibition properties. High levels of antioxidant activities were also reported in the leaf sheaths of dye sorghum extracts [9]. The biocolorants extracted from the dye sorghum leaf sheaths are highly appreciated by consumers in West Africa because they confer a stable bright red colour to foods [10,11]. Traditionally, biocolorants are extracted from dye sorghum leaf sheaths using three artisanal methods of aqueous extraction. These are cool aqueous alkaline extraction, hot aqueous alkaline extraction and hot aqueous extraction. According to Akogou et al. [9], the alkaline extraction methods were the best-performing methods for biocolorant extraction from the leaf sheaths. Cool alkaline extraction is preferred by processors because of its ease of use. However, significant amounts of pigments remained unextracted in the leaf sheath residues. Furthermore, these artisanal extraction methods involve tedious work and do not result in consistent product quality. Many *wagashi* processors and sellers in Benin expressed their need for a ready-to-use and efficient dye sorghum extract to facilitate and allow the profitability of their business [5,10]. In industry, phenolic compounds are extracted using a solvent-extraction procedure [12]. Kayodé et al. [9] reported the efficiency of an ethanol-water mixture in biocolorant extraction from dye sorghum leaf sheaths. Ethanol is a relatively environment-friendly solvent, which additionally benefits from the GRAS (Generally Recognized As Safe) status. Therefore, this study compared three methods to obtain biocolorant from dye sorghum leaf sheaths, namely with 95 % pure ethanol, with *sodabi* (i.e., a local liquor containing 55 % v/v alcohol) and with the traditional cool aqueous alkaline extraction procedure. The aim is to define a sustainable method to extract the red biocolorant from dye sorghum leaf sheaths for dyeing *wagashi*.

## 2 Materials and methods

### 2.1 Plant material

Sun-dried leaf sheaths of a dye sorghum farmer's variety collected from a field in Dassa-Zounmè, Benin, were used for the biocolorant extraction experiments. The leaf sheaths were milled and stored according to Akogou et al. [10].

### 2.2 Biocolorant extraction procedure

**Fig. 2:**
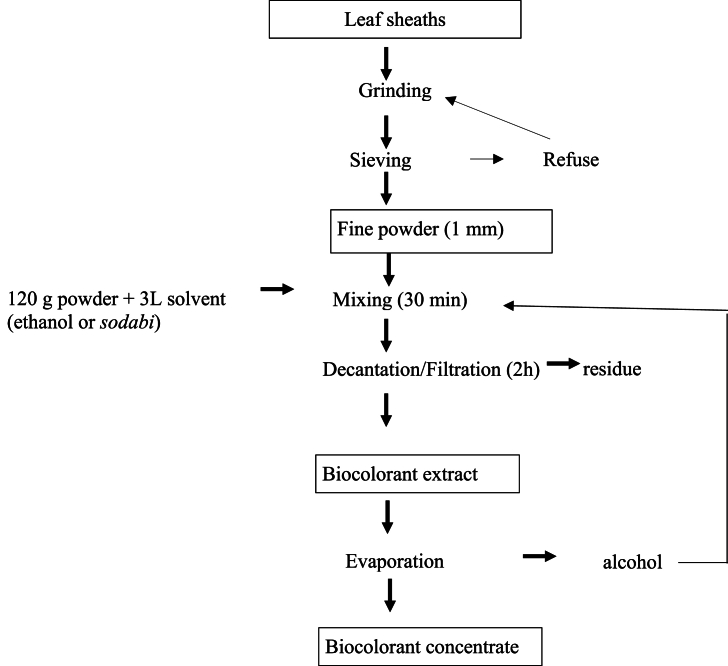
Flow diagram of biocolorant extraction from dye sorghum leaf sheaths.

Leaf sheath powder (120 g) was mixed with 3 L of solvent and stirred for 30 min using a mixer (B20B model, Guangdong Machinery CO., Ltd, China) at a speed of 130 r min^−1^. Extraction solvents included commercial ethanol (95 % v/v) (method A), *sodabi* (a local liquor distilled from palm wine, containing 55 % v/v ethanol) (method B) and water containing 5.4 g L-1 *kanwu*; an alkaline rock salt [10] (method C). The mixture was filtered and allowed to settle for 2 h. The supernatant was collected and ethanol from methods A and B was evaporated at 79 °C for 3 h using a locally purchased pot still evaporator (capacity: 20 L). Ethanol was collected for reuse. After evaporation, the dye obtained from the ethanol extraction (method A) was dissolved in hot water to obtain a volume of 0.5 L. The extractions were carried out in triplicate. The flow diagram of biocolorant extraction from dye sorghum leaf sheaths is shown in Fig. 2.

### 2.3 Wagashi dyeing procedure

Aliquots of 50, 100 and 200 mL of each of the three dye extracts were added to 500 mL water to obtain three concentrations for dyeing the *wagashi* that was freshly prepared by a local processor. The *wagashi* was made using fresh full cow milk following the common *wagashi*-making practices in Benin [11]. The *wagashi* was dyed by soaking three cheese samples of 50 g in each dye solution for 15 min at ambient room temperature (25 °C). Next, the samples were removed and allowed to rest for 5 min at ambient temperature before analysis. Commercial dyed *wagashi* samples were collected from local markets in Cotonou, Dassa- and Parakou for comparative analysis.

### 2.4 Characterization of leaf sheaths

The length and widths of the middle and the basal part of the leaf sheaths were measured for three different samples of 15 sheaths taken at random, making a total of 45 leaf sheaths. The mass of one thousand leaf sheaths (WTS) was determined by weighing one hundred dried leaf sheaths of dye sorghum and multiplying by ten. This measurement was conducted in quadruplicate.

### 2.5 Physico-chemical analyses

#### 2.5.1 Determination of total phenolic compounds (TPC)

TPC were measured according to Kayodé et al. [5].

#### 2.5.2 Determination of 3-deoxyanthocyanidins

3-Deoxyanthocyanidins were assessed by optical density according to Akogou et al. [10].

#### 2.5.3 Colour measurements

Colour parameters were measured according to Akogou et al. [10] with ethanol as reference [13] and characterized following Castellar et al. [14].

### 2.6 Statistical analysis

Data were reported as the mean ± standard deviation, analysed using Minitab 18. Significance was tested by one-way ANOVA with LSD [10].

## 3 Results and discussion

### 3.1 Characterization of leaf sheaths

The average length, middle width and basal width of the leaf sheaths (Table 1) were 30.1, 1.3 and 1.4 cm, respectively. The mass of one thousand leaf sheaths was 2010 g with a water content of 10.2 %. The dimensions of the leaf sheaths corresponded with Kayodé et al. [5]. The values of 43.18; 5.56 and 3.02 were recorded for the *L∗*, *a∗*, and *b∗* colour parameters, respectively (Table 2). The value for the chroma (*C∗*) was 6.34 and for the hue angle (*h*°) 28.53. The total phenolic content of the leaf sheaths was 80.5 mg g^−1^, which aligns with the 82.6 mg g^−1^ previously reported [5,10].

**Table 1:** Dimension characteristics of dye sorghum leaf sheaths.

Sample of dye sorghum leaf sheaths	Dimension		
	Length (cm)	Middle width (cm)	Basal width (cm)
1	32.10	2.10	1.85
2	28.75	1.30	1.84
3	34.10	0.75	1.10
4	30.65	1.50	1.00
5	24.90	1.65	1.29
6	33.25	1.22	1.18
7	32.25	1.74	1.76
8	29.6	0.80	1.66
9	27.2	1.10	1.45
10	28.50	0.95	1.72
Mean	30.13	1.31	1.42
CV	11.64	33.85	26.9

**Table 2:** Physicochemical characteristics of dye sorghum leaf sheaths.

Samples	Colour				Total phenolics (mg/g)	One thousand leaf sheaths weight (g)	Water content (%)
	L∗	a∗	b∗	ΔE			
1	42.50	6.16	3.25	49.54	81.54	2038.16	10.16
2	36.76	8.04	4.41	55.61	78.96	1985.26	12.74
3	41.20	4.95	2.50	50.82	80.32	2007.53	11.68

Mean	43.18	5.56	3.02	50.62	80.52	2010.31	11.52

CV	13.62	24.39	28.41	6.15	01.61	01.32	11.25

### 3.2 Extract characteristics

The obtained quantities of dye extract were 506.2 mL, 533.3 mL and 2645 mL for ethanol extraction, *sodabi* extraction and aqueous alkaline extraction, respectively. The solvent recovery from the pot still evaporator system is 95.6 % for the ethanol extraction procedure and 46.8 % for the *sodabi* extraction procedure. Considering the ethanol grade of the solvent used (95 % v/v for ethanol and 55 % v/v for *sodabi*), the distilling procedure removed most of the ethanol in the extraction solvent. Table 3 shows the colour parameters and the phenolic pigment contents of the various extracts of biocolorant obtained from the three extraction procedures. The results of the analysis of variance on the colour parameters (P > 0) indicated no significant difference between the different extracts in terms of lightness (*L∗*), yellowness index (*b∗*) and hue value (*h*°). However, extracts differ significantly for their redness index *(a∗*) and chroma (*C∗*) (colour intensity) values. Statistical analysis revealed that the chroma values recorded for extracts obtained with the *sodabi* and aqueous alkaline extraction methods were higher than that of the ethanol extract. The values of the hue angle of our extracts (10 < *h*° <15) are comparable to those of certain commercial natural dyes such as hibiscus (*h*° = 13.9) and red carrot concentrate (*h*° = 15.5) [10]. However, there was no perceivable colour difference between the three extracts as indicated by the Total Colour Difference (TCD), which is consistently below 1 (ΔE∗00 < 1). Adekunte [15] reported that differences in perceivable colour can be analytically classified as very distinct (ΔE∗00 > 3), distinct (1.5 < ΔE∗00 < 3) and small (ΔE∗00 < 1.5).

**Table 3:** Colour parameters, total phenolics and 3-deooxyanthocyanidines of various extracts of dye sorghum leaf sheaths.

Extracts	Colour						Total phenolics (mg mL^−1^)	Absorbance (AU mL^−1^)	
	L∗	a∗	b∗	ΔE∗00	C∗	h°		Apigeninidin	Luteolinidin
Ethanol	62.47 ± 0.12a	4.69 ± 0.02b	1.05 ± 0.02a	0.00	4.81 ± 0.02c	12.66 ± 0.32a	21.09 ± 0.29a	1.05 ± 0.02b	1.8 ± 0.33b
Sodabi	62.52 ± 0.18a	4.86 ± 0.06a	1.11 ± 0.03a	0.42	4.98 ± 0.06a	12.85 ± 0.55a	13.42 ± 0.04b	2.84 ± 0.00a	1.9 ± 0.00a
Water + *kanwu*	62.61 ± 0.09a	4.77 ± 0.06b	1.05 ± 0.04a	0.34	4.90 ± 0.00b	12.93 ± 0.13a	1.64 ± 0.14c	0.65 ± 0.00c	0.44 ± 0.00c
Cv	0.11	1.78	3.23	0.19	1.73	1.08	81.3	77.06	59.1

The TPC of the extracts differed significantly. The ethanol extract had the highest amount (21.0 mg mL^−1^), followed by the *sodabi* extract (13.4 mg mL^−1^) and the watery extract (1.6 mg mL^−1^). Indeed, ethanol possesses a strong extraction power as it severely damages the cell membranes of the crushed sheaths allowing high recovery of phenolic pigments [16,17]. The lower TPC value in the *sodabi* extract can be explained by the presence of water in this solvent that contains only 55 % ethanol [18]. Kayodé et al. [5] demonstrated that the phenolic pigments are poorly extracted from dye sorghum leaf sheaths using an aqueous solvent with a low alcohol concentration. Levels of TPC in the two ethanol-containing solvents are also higher than those found by Akogou et al. [10] in the cold and hot aqueous extracts obtained by traditional extraction methods. Concerning the 3-deoxyanthocyanidin content of the extracts, the highest values of apigeninidin and luteolinidine were recorded in the *sodabi* extract (2.84 and 1.9 AU mL^−1^, respectively) followed by the ethanol extract (1.05 and 1.8 AU mL^−1^, respectively). The aqueous alkaline extract had the lowest amounts of apigeninidin and luteolinidine (0.65 and 0.44 AU mL^−1^, respectively). Akogou et al. [10] reported that the alkaline extraction of dye sorghum leaf sheath has a higher recovery of apigeninidin compared to aqueous extraction. The present study revealed that *sodabi* is an even more powerful solvent for the 3-deoxyanthocyanidin extraction. The ethanol extract showed a high colour density and the *sodabi* extract was more reddish than the aqueous alkaline extract.

### 3.3 Dyeing wagashi with the extracts

The extracts from the three types of solvent were used to dye *wagashi*. The colour of the different cheese samples was measured to determine the best colouring extract. *Wagashi* collected from local markets in Cotonou, Dassa and Parakou were used as control. Table 4 shows the colour parameters of the cheeses coloured with the different biocolorant extracts. The lowest values of lightness (*L∗*) were recorded for the *wagashi* coloured with the ethanol extracts while *wagashi* coloured with aqueous alkaline extract presented the highest values. Concerning the redness index (*a∗*), the *wagashi* dyed with ethanol and *sodabi* extracts showed a darker red colour while a lower value of *a∗* was recorded for *wagashi* dyed with the aqueous alkaline extract. The colour intensity for this parameter was dose-dependent, particularly for the *sodabi* and aqueous alkaline extracts. The *a∗* value tended to decrease at higher concentrations of ethanol extract. This phenomenon can be explained by the high extraction power of ethanol. The massive extraction of anthocyanins by ethanol produced an intense red hue, which from a maximum saturation level can give a dark red colour. In this case, a darker-red colouration is perceived. This is further confirmed by the hue angle of the different coloured cheeses. The lowest hue angle values were recorded for *wagashi* coloured with ethanol extracts while the highest values were found in *wagashi* coloured with aqueous alkaline extract. For the three types of extracts evaluated, the hue angle of *wagashi* decreased with an increasing dose of extract in the dyeing solution. The commercial *wagashi* purchased in Dassa and Cotonou were similar in terms of red (*a∗*) and yellow (*b∗*) colour intensities but were less coloured than commercial *wagashi* from Parakou. Overall, the commercial *wagashi* showed lower redness index values compared to *wagashi* coloured with ethanol and *sodabi* extracts while the *wagashi* dyed with the aqueous alkaline extract exhibited the lowest *a∗* values.

**Table 4:** Colour parameters of *wagashi* dyed with various extracts of dye sorghum leaf sheaths.

Extracts	Dose (mL/L)	L∗	a∗	b∗	ΔE	C∗	h°
*Wagashi* dyed with ethanol extract	100	77.53 ± 0.22e	35.83 ± 0.87ab	21.54 ± 0.16d	38.33 ± 0.28b	41.45 ± 0.31b	31.31 ± 0.20f
	200	75.25 ± 0.82f	34.60 ± 0.89ab	19.44 ± 0.57e	36.27 ± 0.94c	39.69 ± 1.05c	29.32 ± 0.18f
	400	68.90 ± 0.05g	30.57 ± 0.30c	13.4 ± 0.22f	29.75 ± 0.31g	33.38 ± 0.32d	23.67 ± 0.34g
*Wagashi* dyed with sodabi extract	100	86.45 ± 0.42b	19.78 ± 1.72d	23.73 ± 0.21c	31.59 ± 0.62d.e	30.91 ± 0.93f	50.23 ± 2.74d
	200	82.03 ± 0.59c	34.13 ± 0.54b	23.89 ± 0.57c	39.69 ± 0.48a	41.66 ± 0.52a.b	35.00 ± 0.80^e^
	400	78.43 ± 0.21d	36.27 ± 0.46a	22.18 ± 0.58d	39.6 ± 0.68a	42.52 ± 0.69a	31.44 ± 0.35f
*Wagashi* dyed with water + kanwu extract	100	90.91 ± 0.25a	1.49 ± 0.92g	26.35 ± 0.26b	30.87 ± 0.35ef	26.41 ± 0.22g	86.73 ± 2.04a
	200	90.26 ± 0.27a	3.38 ± 0.90f	26.75 ± 0.14ab	30.66 ± 0.24f	26.97 ± 0.11g	82.79 ± 1.92b
	400	86.51 ± 0.55b	17.36 ± 1.88e	27.20 ± 0.60a	32.31 ± 0.26d	32.31 ± 0.55e	57.49 ± 3.35c
*Wagashi* from local markets	Cotonou	73.15 ± 0.41h	23.64 ± 0.62h	14.02 ± 0.55f	32.55 ± 0.88d	29.48 ± 0.99h	35.68 ± 0.57^e^
	Dassa	79.86 ± 0.21d	24.51 ± 0.40h	15.33 ± 0.32f	30.31 ± 0.49f	30.33 ± 0.52i	37.16 ± 0.52i
	Parakou	76.12 ± 0.12f	30.70 ± 0.84c	17.38 ± 0.92^e^	37.76 ± 1.05bc	37.16 ± 0.31j	35.42 ± 0.98^e^

**Fig. 3:**
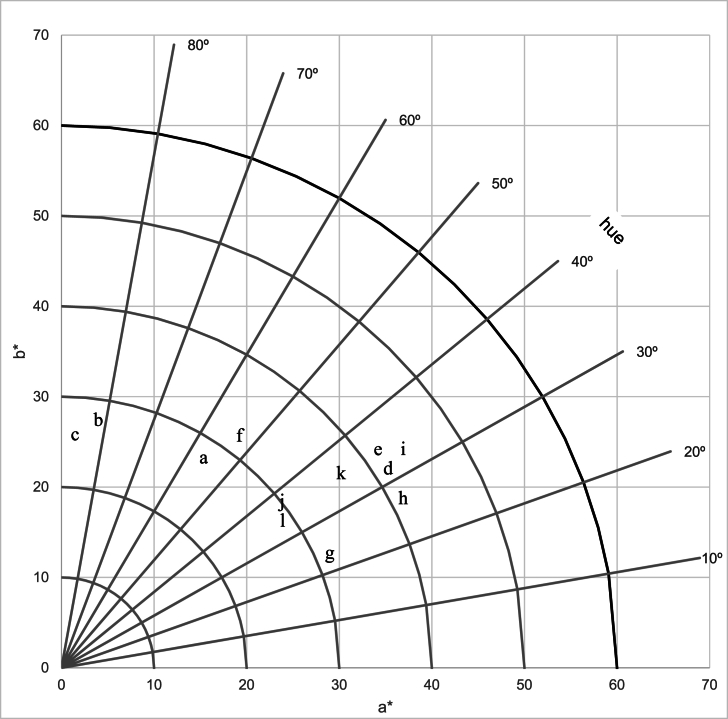
The chromaticity diagram of *wagashi* dyed with extracts at various concentrations. *Wagashi* dyed with aqueous alkaline extract: 400 mL L^−1^ (a), 200 mL L^−1^ (b), 100 mL L^−1^ (c), *Wagashi* dyed with *sodabi* extract: 400 mL L^−1^ (d), 200 mL L^−1^ (e), 100 mL L^−1^ (f), *Wagashi* dyed with ethanol extract: 400 mL L^−1^ (g) 200 mL L^−1^ (h), 100 mL L^−1^ (i), Control *wagashi* from Dassa market (J), Parakou market (k) and Cotonou market (l).

**Fig. 4:**
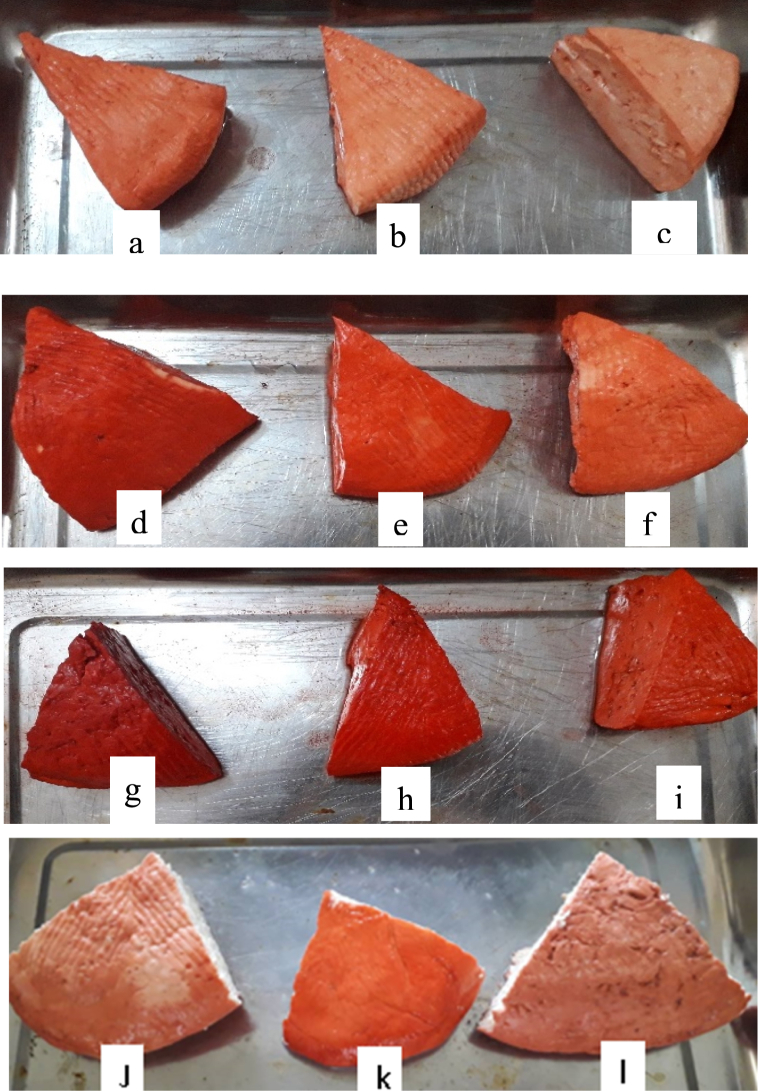
Surface colour of *wagashi* dyed with extracts at various concentration: Dyed with aqueous alkaline extract: 400 mL L^−1^ (a), 200 mL L^−1^ (b), 100 mL L^−1^ (c), Dyed with *sodabi* extract: 400 mL L^−1^ (d), 200 mL L^−1^ (e), 100 mL L^−1^ (f), Dyed with ethanol extract: 400 mL L^−1^ (g) 200 mL L^−1^ (h), 100 mL L^−1^ (i), Control *wagashi* from Dassa market (J), Parakou market (k) and Cotonou market (l).

Fig. 3 shows the chromaticity diagram of the various dyed samples. The twelve samples of *wagashi* (Fig. 4) could be grouped into 5 groups based on their chroma and hue values. Group 1 (Fig. 4g) includes only the *wagashi* coloured with ethanol extract at a dose of 400 mL L^−1^. This is the most colourful and intense *wagashi*. The second group (Fig. 4i, h, e, d, k) includes *wagashi* dyed with ethanol extract at the doses of 100 and 200 mL L^−1^, *wagashi* dyed with *sodabi* extract at the doses of 200 and 400 mL L^−1^ and the commercial sample from Parakou. These *wagashi* samples are less red than the one in the first group but darker red than the remaining *wagashi* samples. In the third group (Fig. 4j, l), we find the commercial *wagashi* samples from Dassa and Cotonou with less intense red colours than those of the first two groups. The fourth group (Fig. 4a, f) combines *wagashi* dyed with aqueous alkaline extract at a dose of 400 mL L^−1^ and *wagashi* dyed *sodabi* extract at a dose of 100 mL L^−1^. The last group (Fig. 4c, b) comprises the *wagashi* dyed with aqueous alkaline extract at doses of 100 and 200 mL L^−1^, which are the least colourful *wagashi* samples.

## 4 Conclusion

We developed a sustainable and efficient process for biocolorant extraction from dye sorghum leaf sheaths in view to alleviate the daily dyeing tasks of thousands of women selling *wagashi* in Benin. In terms of pigment extraction, commercial ethanol proved to be the best-extracting solvent followed by *sodabi* while the aqueous alkaline solvent poorly extracted the pigments. Considering the colouring power of the extracts, and the cost and availability of the solvent, *sodabi* is to be the preferred solvent of the three types of solvents tested. The application of *sodabi* extract at a dose of 200 mL L^−1^ is recommended for dye sorghum leaf sheath extraction for *wagashi* dyeing for the Beninese market.

## CRediT authorship contribution statement

**Oscar Bienvenu Oloudé Odouaro:** Writing – original draft, Software, Methodology, Investigation, Formal analysis, Data curation. **Adéchola Pierre Polycarpe Kayodé:** Writing – review & editing, Writing – original draft, Validation, Supervision, Resources, Project administration, Methodology, Funding acquisition, Conceptualization. **Médérice Sènanmi Behanzin:** Writing – original draft, Software, Methodology, Formal analysis, Data curation. **Martinus J. Rob Nout:** Writing – review & editing, Validation, Supervision, Resources, Methodology, Funding acquisition, Conceptualization. **Anita R. Linnemann:** Writing – review & editing, Validation, Supervision, Resources, Project administration, Methodology, Funding acquisition, Conceptualization.

## Data availability statement

All the relevant data are included in the manuscript. No separate repository is attached.

## Declaration of competing interest

The authors declare that they have no known competing financial interests or personal relationships that could have appeared to influence the work reported in this paper.
